# Genome analysis of third-generation cephalosporin-resistant *Escherichia coli* and *Salmonella* species recovered from healthy and diseased food-producing animals in Europe

**DOI:** 10.1371/journal.pone.0289829

**Published:** 2023-10-26

**Authors:** Marlène Sauget, Alban K. Atchon, Benoît Valot, Farid El Garch, Anno de Jong, Hilde Moyaert, Didier Hocquet, on behalf of the EASSA and VetPath Study Group

**Affiliations:** 1 Service D’hygiène Hospitalière, Centre Hospitalier Universitaire, Besançon, France; 2 Bioinformatique et Big Data au Service de la Santé, UFR Santé, Université de Bourgogne Franche-Comté, Besançon, France; 3 UMR 6249 CNRS Chrono-Environnement, Université de Bourgogne Franche-Comté, Besançon, France; 4 Vétoquinol SA, Global Drug Development Center, Lure, France; 5 EASSA and VetPath Study Group, CEESA, Brussels, Belgium; 6 Zoetis Belgium SA, Veterinary Medicine Research and Development, Zaventem, Belgium; Cornell University College of Veterinary Medicine, UNITED STATES

## Abstract

The animal reservoir of Enterobacterales producing Extended-Spectrum-β-Lactamases (ESBL) and plasmid-borne cephalosporinases (pAmpC) is a global concern. Using genome data, we analyzed a population of *Escherichia coli* and *Salmonella* species resistant to third-generation cephalosporins (3GC-R) recovered from healthy food animals (HA) and diseased food animals (DA) across Europe. Among the isolates collected from HA (n = 4,498) and DA (n = 833) in up to twelve European countries, 62 (1.4%) and 45 (5.4%) were 3GC-R, respectively. The genomes of these 3GC-R 107 isolates were sequenced to identify *bla*_ESBL_ and *bla*_AmpC_, sequence types (STs), virulence-associated genes, and *Salmonella* serovars. We also assessed their population structure using core genome multilocus sequence typing. The 78 3GC-R *Escherichia coli* originated from poultry (n = 27), swine (n = 26), and cattle (n = 25). Almost all (n = 77; 98.7%) harbored at least one *bla*_ESBL_ or *bla*_AmpC_, with *bla*_CTX-M-1_ predominating. We identified 51 STs, with ST10 and ST101 being the most frequent. The population of 3GC-R *E*. *coli* was polyclonal. The 29 3GC-R *Salmonella* spp. were mostly retrieved from healthy broiler (96.5%). *bla*_CMY-2_ dominated in this population. We found two clusters of CMY-2-producing *Salmonella* spp. in Germany: one with 15 isolates of *S*. Heidelberg isolates and another with six *S*. Minnesota, all of them with *bla*_CMY-2_. Our results confirm the low prevalence of 3GC-R *E*. *coli* and *Salmonella* spp. in HA and DA. *bla*_CTX-M-1_ was dominating in a highly diverse population of *E*. *coli*. 3GC-R *E*.*coli* isolated from HA and DA were genetically unrelated, with high clonal diversity suggesting multiple origins of contamination. This contrasted with the clonal population of 3GC-R *Salmonella* spp. in which *bla*_CMY-2_ dominated through two dominant serovars in this collection.

## Introduction

The wide dissemination of Enterobacterales producing Extended-Spectrum-β-Lactamases (ESBL) and plasmid-borne cephalosporinases (pAmpC) is a global health concern [1–3]. The worldwide distribution of genes encoding ESBL or pAmpC has evolved over the time [4]. In the last decade, ESBL- and pAmpC-producing Enterobacterales have spread in humans in hospitals and community, in animals, and in the environment [5–7]. Understanding the circulation of these resistant pathogens among these reservoirs requires a One Health approach [8, 9].

The impact of resistance to cephalosporins in therapeutic success can be particularly severe when affecting vulnerable patients, such as children and immunocompromised individuals. Difficult-to-treat human infections are commonly associated with multidrug-resistant bacteria resistant to antimicrobials that are often considered first-line drugs for empirical therapy of severe infections such as β-lactam antibiotics. *Escherichia coli* is one of the leading pathogens associated with resistance, with *E*. *coli* carrying genes encoding ESBL or AmpC being the antibiotic-resistant pathogen with the higher burden for humans [10]. Global dissemination and increased prevalence of ESBL and AmpC in Enterobacterales is a public health concern of particular relevance, since resistance to β-lactams limits the choice of effective antimicrobial therapies. An increased prevalence of ESBL and AmpC has been observed in the human gut microbiota in both healthy and diseased members in hospitals and the community. In parallel with the increased incidence in humans, Enterobacterales producing ESBL or AmpC are frequently found in livestock, the food chain, and companion animals, making domestic animals the reservoir and vehicle for the spread of these resistant pathogens. Although it is now known that human-to-human transmission is the primary route of community-acquired ESBL- or AmpC-producing *E*. *coli* [11], a proportion of human infections with these resistant pathogens still originate from food-producing animals [12]. The understanding of the dynamics of the transmission of genes encoding ESBL or AmpC requires the exploration of the size and the diversity of the reservoir of resistant pathogens in food-producing animals.

*Escherichia coli* and *Salmonella* spp. (Ec/Salm) are two of the most common bacterial groups producing ESBL and pAmpC, with a few clones playing a major role in dissemination of the corresponding genes [1, 13, 14]. ESBL/pAmpC encoding genes spread readily between bacteria due to their presence on mobile genetic elements, including plasmids and transposons [3, 15, 16]. The most common ESBLs belong to CTX-M, TEM, and SHV families. Besides, CMY enzymes, among which CMY-2 dominates, are the most frequent pAmpC [13, 14, 17, 18]. The nature and the prevalence of the ESBL/pAmpC variants differ between human and animal reservoirs, further complicating the identification of potential sources [14, 17]. Thus, *E*. *coli* ST131 carrying *bla*_CTX-M-15_ spread among humans in Europe, while they were rare in food-producing animals [8, 19]. This contrasts with *bla*_CTX-M-1_, more frequent in animals than in humans [4, 13–14, 20, 21]. Recently, *bla*_SHV-12_ and *bla*_CTX-M-15_ have been found frequently in food-producing and companion animals, respectively [6, 7]. In their extensive analysis of the distribution dynamics of ESBL/AmpC genes in *E*. *coli* from Europe and North America, Zamudio *et al*. [22] found contrasting temporal dynamics. AmpC initially dominated in Canada and USA in farm animals and humans, and emerged later in Europe. In contrast, ESBLs were initially common in animals from Europe and later emerged in North America. There was variation in the major ESBL/AmpC gene frequency between countries and host sources, and the main mechanism, clonality and/or plasmids, responsible for the dissemination of these genes. International travels and trades have likely influenced the global dissemination of these genes [22]. In this context, global trends in the prevalence of ESBL/AmpC in Enterobacterales in animals are difficult to assess due to large variations between countries and limited data over time. For Europe, however, some statements can be made. The prevalence of ESBL/AmpC in commensal Enterobacterales in livestock is generally low, although across some European countries moderate levels of resistance have been observed in broilers [14]. However, recently the proportion of ESBL/AmpC in *E*. *coli* has declined in Europe as a possible consequence of the reduction in antibiotic use [14, 23, 24]. Indeed, the overall sales of antimicrobials including cephalosporins in veterinary medicine in Europe have approximately halved from 2010 to 2019 [25]. In contrast, antibiotic consumption in human medicine remains approximately constant.

The animal reservoir is complex with different sectors (*i*.*e*. wild animals, companion animals, food-producing animals) at different health states (healthy or diseased). The continuous monitoring of the food-producing animals sector is crucial to evaluate the size and the diversity of this reservoir of antimicrobial resistance genes. This applies to the ESBL/pAmpC reservoirs of both diseased and healthy animals. With respect to diseased animals, the identification of ESBL/pAmpC-producing strains is important for the option to treat diseased animals as well as for the risk of transmission to humans by direct contact to humans or by slaughter. Concomitantly the size of the ESBL/pAmpC reservoir in healthy animals is of great relevance for public health since they can contaminate human populations. When infections caused by these bacteria occur, it can be more challenging and difficult to treat if antibiotic classes are used in both animal and human medicines. In this context, we used genome-based data to determine and compare the population structure of Ec/Salm isolates resistant to third-generation cephalosporins (3GC-R) recovered from both healthy (HA) and diseased (DA) cattle, swine, and chickens in Europe, and further identify genes encoding ESBL and pAmpC.

## Materials and methods

### Bacterial isolates

The European Antimicrobial Susceptibility Surveillance in Animals program (EASSA) monitors the antimicrobial susceptibility of zoonotic and commensal bacteria in healthy food-producing animals at slaughter throughout Europe [26]. In the same way, the European Antimicrobial Susceptibility Surveillance for food animal pathogens program (VetPath) examines the antimicrobial susceptibility of major disease-causing bacterial pathogens in food-producing animals across Europe [26]. The EASSA and VetPath projects include major countries of production of cattle, swine, and broiler chickens in Europe. With these two monitoring programs, isolates from HA and DA were collected from 2015 to 2018 in twelve European countries (Belgium, Czech Republic, Denmark, France, Germany, Hungary, Italy, The Netherlands, Poland, Spain, Switzerland, and the United Kingdom).

For the EASSA program, samples were collected from at least 4 abattoirs per country and from each herd or flock, only one animal was randomly selected for sampling. To obtain a collection of epidemiologically unrelated strains, only one isolate per sample was retained. In this program, the *E*. *coli* isolates were recovered from approximately 200 samples of intestinal contents per country and animal species, whereas *Salmonella* isolates were retrieved from national surveys in two countries (France, Hungary), from carcass broiler samples in Germany, and from monitoring samples in Spain. Ec/Salm isolates were recovered from samples with standard procedures using MacConkey agar (Oxoid, Thermofisher Scientific) and Semisolid Rappaport Vassiliadis Medium (Micromedia, Edwards).

For the VetPath program, Ec/Salm isolates were retrieved from rectal, fecal, or visceral samples taken from cattle or swine with acute clinical signs of digestive diseases. Standard culture media (see above) were used to isolate Ec/Salm. For post-partum dysgalactia syndrome (PPDS) in swine, formerly designated as mastitis-metritis-agalactia (MMA), milk samples or uterine swabs were taken. For broilers with respiratory or septicemia infections, visceral samples or swabs from lung tissue were retrieved. Only one sample per outbreak per farm was included from each herd sampled [26]. In this VetPath program, number of Enterobacterales isolates varied from 15 to 184 isolates per country and from 1 to 78 per animal species and country.

### Bacterial identification and antimicrobial susceptibility testing

Bacterial species were identified by matrix-assisted laser desorption/ionization time-of-flight mass spectrometry (MALDI-ToF MS) (Microflex; Bruker Daltonics, Bremen, Germany) according to the manufacturer’s recommendations. *Salmonella* isolates were serotyped according to the Kaufmann-White scheme by a standardized procedure (*i*) either locally for isolates from France and Hungary, (*ii*) or by the National Institute for Public Health and the Environment (RIVM, Bilthoven, The Netherlands) for isolates from Germany and Spain. Minimal inhibitory concentrations (MICs) of cefotaxime (CTX) and ceftazidime (CAZ) were determined by broth dilution, which were interpreted according to the European Committee on Antimicrobial Susceptibility Testing (EUCAST) breakpoints (www.eucast.org: version 11.0, January 2021). ESBL production was detected with synergy test (www.eucast.org: version 2.0, July 2017). 3GC-R isolates (i.e. with MIC > 2 mg/L of CTX and/or MIC > 4 mg/L of CAZ) were selected for whole-genome sequencing. MICs of the antimicrobials agents were determined in the regular monitoring programs of EASSA and VetPath. The antimicrobials tested belong to the major antibiotic classes, i.e. penicillins, cephalosporins, fluoroquinolones, phenicols, polymyxins, aminoglycosides, carbapenems, folate pathway inhibitors, tetracyclines, and glycylcyclines [26]. Multidrug resistance (MDR) of an isolate was defined as clinical resistance to at least one agent in three or more antimicrobial classes [27].

### Whole-genome sequencing and analysis

Bacterial deoxyribonucleic acid (DNA) was extracted with QIAamp DNA mini kit (Qiagen) and quantified using spectrophotometry analysis (NanoDrop 2000, ThermoScientific). Libraries were prepared with Illumina Nextera XT. We sequenced the genomes with 2 x 150-bp reads on Illumina NextSeq 500 at high-output mode (v2.5). Raw read coverage was homogenized at 80x by subsampling with a homemade script. Then, genomes were assembled *de novo* using SPAdes (v3.13.1) with careful mode. Low coverage contigs (<1x) were removed. Indicators assessed the quality of the assembly (number of contigs per sample, minimal coverage). Resistance, virulence, and plasmids determinants of the 3GC-R isolates were determined from the sequences of the whole-genomes analyses with online resources present at the Center for Genomic Epidemiology (genomicepidemiology.org). For this purpose, genes of ResFinder, VirulenceFinder, and PlasmidFinder database were manually searched after download on the assembly genome using BLAT (BLAST-Like Alignment Tool) software with 80% of coverage and 90% identity thresholds [28]. Resfinder databases identified genes encoding ESBL and pAmpC along with the resistance determinants to all antimicrobials (S4 and S5 Tables).

Core genome multilocus sequence typing (cgMLST) was calculated from allelic comparison of genes and the sequence types (STs) were identified with an in-house tool (github.com/bvalot/pyMLST). We built phylogenetic trees from the distances calculated on the core genome genes using Neighbor-Joining algorithm with Grapetree software [29]. Phylogenetic tree figure were built on the iTOL (Interactive Tree Of Life) website [30]. We analysed the cgMLST by alignment of genes shared by ≥ 95% (core genome) of the isolates against the genes in the multilocus sequence typing alleles assigned to each species in the cgMLST database. From the identified genes, we calculated the genetic distance matrices to generate the trees with the Neighbor-Joining algorithm.

All paired-end reads are available under Bioproject accession number PRJNA848630.

### Statistical analysis

Because this study is descriptive in nature and the low number of isolates per country or animal host frequently prevented meaningful statistical analyses for *Salmonella* spp., we limited the statistical analyses to *E*. *coli* and focused on the comparison of ESBL/AmpC types and differences between the HA and DA populations. The comparison of categorical variables was performed using the Pearson’s two-sided Chi-squared test or Fisher’s exact test, as appropriate. Data were analyzed by using the open Epi^R^ software. In all tests, a *P* value of ≤0.05 was considered as significant.

## Results

### Bacterial collection and antimicrobial susceptibility

During the four-year study, we found that 1.4% (n = 62 out of 4,498) and 5.4% (n = 45 out of 833) of the Enterobacterales recovered from HA and DA were 3GC-R, respectively (Table 1). The S1 Table (78 *E*. *coli*) and S2 Table (29 *Salmonella* spp.) indicate the ESBL/pAmpC type of enzymes and country distribution of these 107 isolates. The following paragraph describes the distribution of 3GC-R *E*. *coli* and *Salmonella* isolates between countries, animal species, and their health status (HA or DA).

**Table 1:** Enterobacterales distribution according to animal species and country for the VetPath and EASSA program.

	Total number of isolates (number of 3GC-R isolates)			
***E*. *coli* EASSA**	**Calf/Cattle**	**Swine**	**Broilers**	**Total**
France	390 (0)^a^	214 (1)	172 (4)	776 (5)
Belgium	208 (0)	-^b^	-	208 (0)
Germany	171 (0)	210 (7)	199 (0)	580 (7)
Hungary	-	-	220 (9)	220 (9)
Italy	185 (2)	-	-	185 (2)
The Netherlands	-	216 (0)	216 (2)	432 (2)
Poland	203 (0)	-	-	203 (0)
Spain	-	201 (1)	199 (7)	400 (8)
United Kingdom	201 (0)	204 (0)	201 (1)	606 (1)
Total	1377 (2)	1045 (9)	1207 (23)	3610 (34)
***Salmonella* EASSA**	**Calf/Cattle**	**Swine**	**Broilers**	**Total**
France	16 (0)	204 (0)	110 (0)	330 (0)
Germany	1 (0)	9 (0)	259 (26)	269 (26)
Hungary	-^a^	19 (0)	203 (2)	222 (2)
Spain	-	64 (0)	3 (0)	67 (0)
Total	17 (0)	296 (0)	575 (28)	888 (28)
**Enterobacterales VetPath**	**Calf/Cattle**	**Swine**	**Broilers**	**Total**
France	76 (8)	67 (15)	41 (2)	184 (25)^b^
Belgium	37 (0)	61 (1)	23 (2)	121 (3)
Czech Republic	15 (0)	- ^a^	-	15 (0)
Denmark	-	39 (0)	-	39 (0)
Germany	29 (4)	61 (0)	1 (0)	91 (4)
Italy	63 (8)	-	-	63 (8)
The Netherlands	37 (0)	54 (0)	-	91 (0)
Poland	-	33 (0)	-	33 (0)
Spain	-	77 (0)	-	77 (0)
Switzerland	19 (0)	-	-	19 (0)
United Kingdom	22 (4)	78 (1)	-	100 (5)
Total	298 (24)	470 (17)	65 (4)	833 (45)^c^

^a^In France 192 isolates were recovered from beef cattle samples and 198 isolates from veal calf samples.

^b^-: indicates that no isolates collected.

^a^–indicates that no isolates were collected.

^a^–: indicates that no isolates were collected.

^b^One bovine *Salmonella* strain is included. Number of *E*. *coli* is 183 (24).

^c^One bovine *Salmonella* strain is included. Number of *E*. *coli* is 832 (44).

France (n = 29, 37.2%) was the main source of 3GC-R *E*. *coli* isolates followed by Germany (n = 11, 14.1%), Italy (n = 10, 12.8%), Hungary (n = 9, 1.5%), Spain (n = 8, 10.3%), UK (n = 6, 7.7%), Belgium (n = 3, 3.8%), and the Netherlands (n = 2, 2.6%) (S1 Table). More specifically, 34 3GC-R *E*. *coli* isolates were isolated from HA in 7 out of 9 countries included (except for Belgium and Poland) and 44 isolates from DA in 5 out of 11 countries, mostly raised in France (n = 24, 54.5%) (Table 1). Of the individual countries, a broad variation of 3GC-R *E*. *coli* is apparent for both HA and DA (Table 2). HA 3GC-R *E*. *coli* mainly originated from broilers (n = 23, 67.6%), whereas DA 3GC-R *E*. *coli* mostly originated from cattle (n = 23, 56.4%) and swine (n = 17, 38.6%) (Table 1). For DA, 3GC-R *E*. *coli* were mainly isolated from diarrhea cases (n = 34, 77.3%).

**Table 2:** Overview of 3GC-R *E*. *coli* (in percentages) according to animal health status and country for the VetPath and EASSA program.

	BE	FR	CZ	DK	DE	IT	NL	PL	ES	CH	UK
Diseased animal (VetPath)	2.5	13.1	0.0	0.0	4.4	12.7	0.0	0.0	0.0	0.0	5.0
Healthy animal (EASSA)	0.0	0.6	-	-	1.2	0.1	0.5	0.0	2.0	-	0.2
Total	0.9	3.0	-	-	1.6	4.0	0.4	0.0	1.7	-	0.8

-: a dash indicates country not included in the programme.

All 3GC-R *Salmonella* spp. HA (n = 28) were sampled from broiler mainly raised in Germany (n = 26, 92.6%) (S2 Table). Only one *Salmonella* Typhimurium DA was isolated from a calf diarrhea case in France.

Clinical breakpoints had been set for all compounds tested in the EASSA program, enabling the identification of MDR isolates from healthy animals. The proportion of MDR isolates amounted to 88.2% (n = 30) for 3GC-R *E*. *coli*, and 92.9% (n = 26) for 3GC-R *Salmonella* spp. (S3 Table). Unfortunately, the proportion of MDR isolates from diseased animals could not be assessed since the antibiotic compounds tested were mostly specific to veterinary medicine, with no published clinical breakpoints. However, based on the presence of the genes responsible for the resistance to the nine major classes of antimicrobials used in veterinary medicine (*i*.*e*. β-lactams, fluoroquinolones, phenicols, tetracyclines, diaminopyrimidines, sulfonamides, aminoglycosides, lincosamides, macrolides, polymyxins), we found that the majority of the ESBL/AmpC producing isolates also harbored resistance genes against aminoglycosides, phenicols, sulfonamides, macrolides and tetracyclines. Hence, 84.1% (n = 37) of *E*. *coli* isolates were MDR (*i*.*e*. harbored resistance genes against at least three different classes of antibiotics) (S4 Table). The only 3GC-R *Salmonella* spp. of the VetPath collection carrying multiple resistance genes could also be considered as MDR (S5 Table). In addition, genome sequencing of the 45 VetPath 3GC-R isolates revealed that (*i*) none of them harbored carbapenemase-encoding genes and (*ii*) eight isolates (17.7%) had a colistin resistance gene *mcr-1* (6 *E*. *coli* isolates), *mcr-3* (1 *Salmonella* isolate), or *mcr-4* (1 *E*. *coli* isolate) (S4 and S5 Tables). All eight *mcr*-carrying isolates harbored genes encoding resistance to aminoglycosides, macrolides (except the Salmonella isolate), phenicols, sulfonamides, diaminopyrimidines and tetracyclines (S4 and S5 Tables).

### Resistance determinants to third-generation cephalosporins

**Fig 1:**
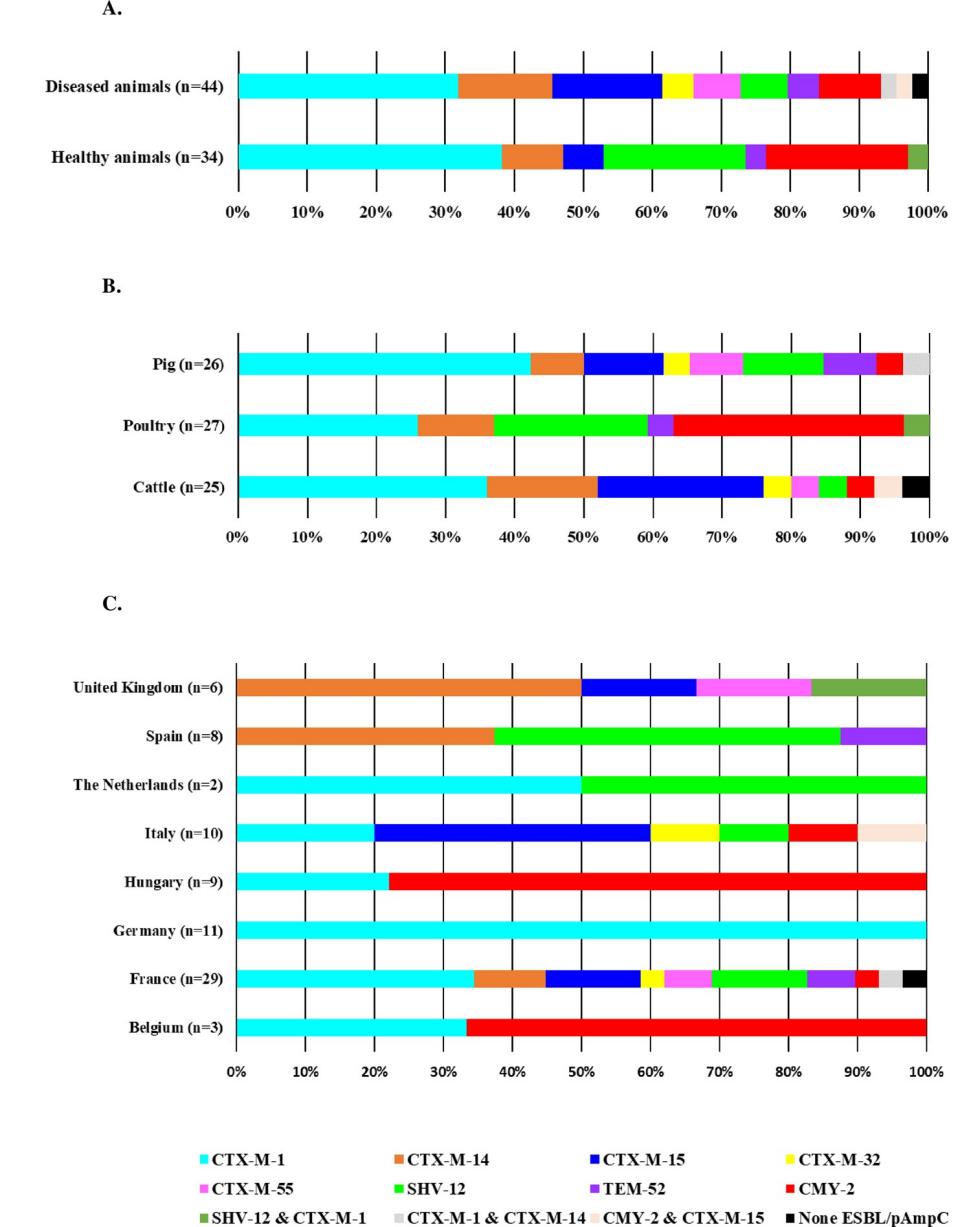
ESBLs/pAmpC distribution among third-generation cephalosporin resistant *E. coli* (n = 78) isolated from healthy and diseased food-producing animals between 2015 and 2018 in Europe.

**Fig 2:**
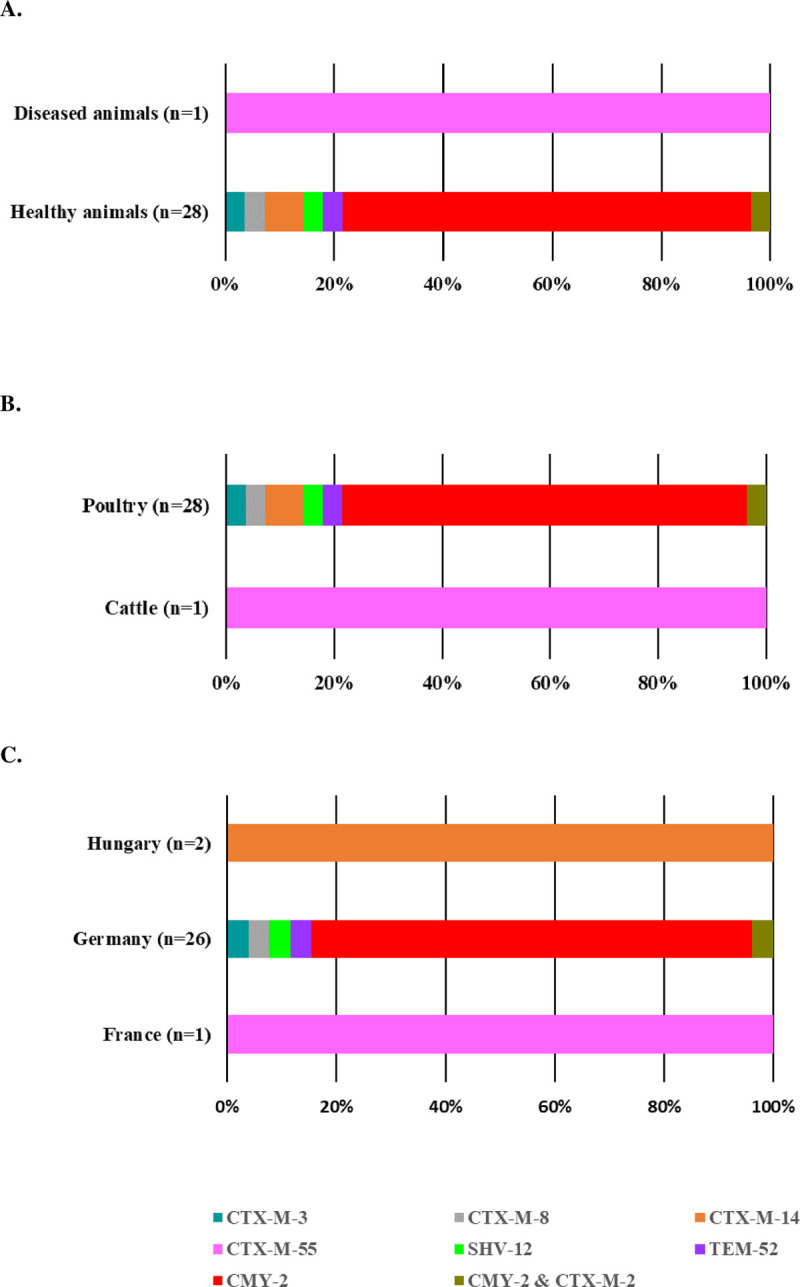
ESBLs/pAmpC distribution among third-generation cephalosporin resistant *Salmonella* spp. (n = 29) isolated from healthy and diseased food-producing animals between 2015 and 2018 in Europe.

In total, 106 out of the 107 (99.1%) Ec/Salm sequenced harbored either genes encoding ESBL or pAmpC (Figs 1 and 2). The one *E*. *coli* isolate without ESBL/AmpC likely overexpressed its chromosomal AmpC cephalosporinase due to mutations. We identified eleven (eight for *E*. *coli*; eight for *Salmonella* spp.) different genes encoding ESBL or AmpC enzymes.

Among the 3GC-R *E*. *coli* isolates, 34 *E*. *coli* HA (n = 34, 100%) and 43 *E*. *coli* DA (n = 43, 97.7%) harbored one or more ESBL/pAmpC encoding gene (Fig 1A). Overall, 36 isolates out of 78 (46.2%) carried a *bla*_CTX-M_ of group 1 (27 *bla*_CTX-M-1_ and 9 *bla*_CTX-M-15_). *bla*_CTX-M-1_ dominated (n = 27, 34.6%) in both HA (n = 13, 38.2%) and DA (n = 14, 31.8%), followed by *bla*_CMY-2_ (n = 11, 14.1%), *bla*_SHV-12_ (n = 10, 12.8%), *bla*_CTX-M-14_ (n = 9, 11.5%), and *bla*_CTX-M-15_ (n = 9, 11.5%). Three isolates exhibited combinations of two different ESBL/pAmpC genes (Fig 1). *bla*_CTX-M-1_ dominated in cattle and swine (n = 9, 36.0% and n = 11, 42.3%, respectively) followed by several other ESBL encoding genes (Fig 1B). In contrast, *bla*_CMY-2_ dominated in broilers (n = 9, 33.4%) and was retrieved mostly in healthy broilers from Hungary (Fig 1C).

Considering the three animal species together (cattle, swine, and poultry), the distribution of ESBL/pAmpC was not different (*P*>0.05 for each ESBL/pAmpC variable) between the HA and DA populations. Analysis of the distribution of the enzymes between animal species revealed that *bla*_CTX-M-1_ differentially distributed between healthy and diseased swine (*P* = 0.0127). Considering the HA and DA populations together, we found that the distribution of *bla*_CTX-M-15_ and *bla*_CMY-2_ differed between cattle and poultry (*P =* 0.0174 and *P =* 0.0159, respectively) and for *bla*_CMY-2_ between poultry and swine (*P =* 0.0134).

All 29 3GC-R *Salmonella* spp. harbored one or two ESBL/pAmpC encoding genes (Fig 2). *bla*_CMY-2_ gene dominated in the *Salmonella* spp. HA isolated from poultry raised in Germany (n = 21, 75.0%) whereas the only *Salmonella* Typhimurium DA harbored a *bla*_CTX-M-55_. The two *S*. Infantis isolates from Hungary carried both *bla*_CTX-M-14._ No ESBL/pAmpC encoding genes were detected in *Salmonella* spp. isolates from France and Spain (n = 397) (Table 1).

#### Population structure of the 3GC-R E. coli and *Salmonella* spp

**Fig 3:**
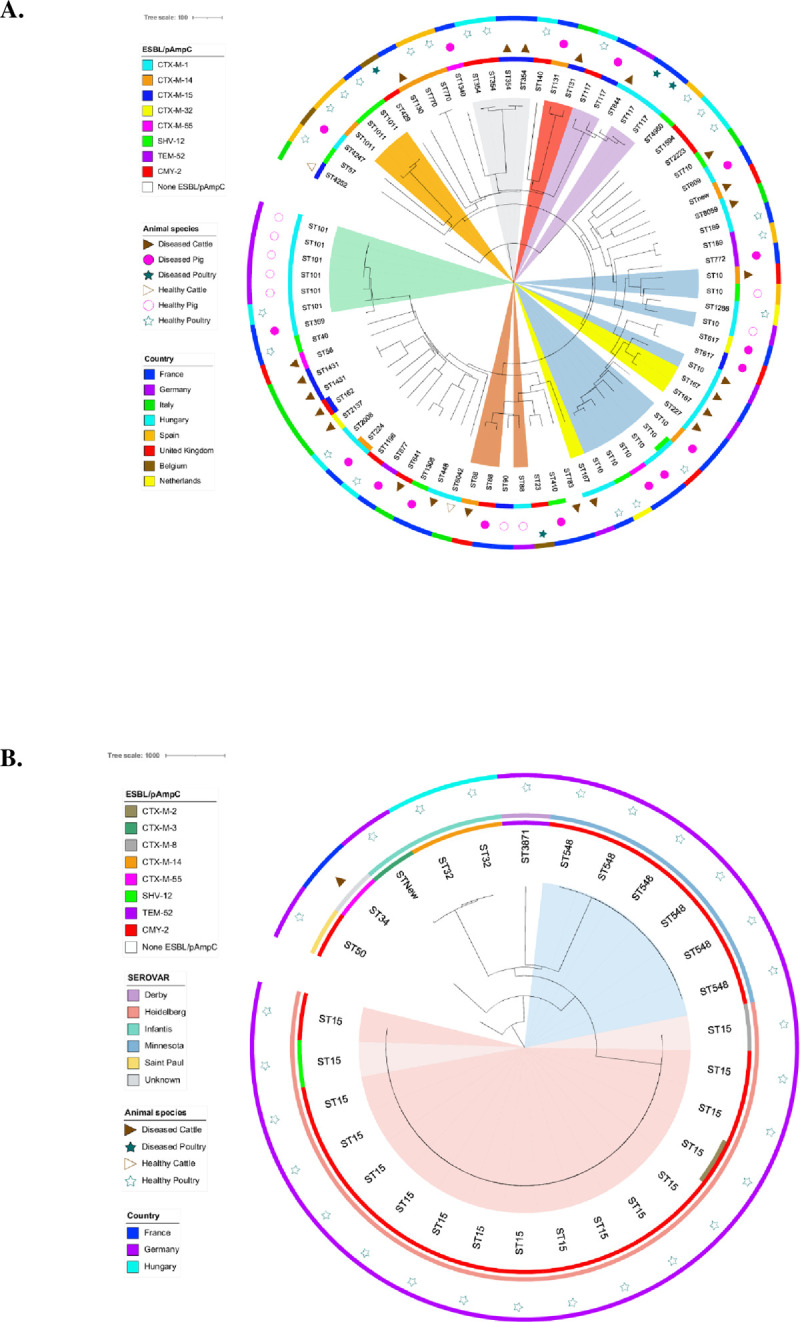
Population structure of the third-generation cephalosporin resistant *E*. *coli* (n = 78) and *Salmonella* spp. (n = 29) isolated from healthy (HA) and diseased (DA) food-producing animals between 2015 and 2018 in Europe.

*E*. *coli* cgMLST revealed a high genetic variability with a polyclonal distribution. Hence, 20 STs and 31 STs were identified for 3GC-R *E*. *coli* HA and DA, respectively. Overall, 35 STs were represented by a single isolate (S1 Table). In the whole *E*. *coli* collection, the most frequent STs were ST10 (n = 10), ST101 (n = 6), ST117/ST354 (n = 4 each), and ST88/ST167/ST1011 (n = 3 each) (S1 Table). Whereas Fig 3A shows the phylogenetic tree built with all HA and DA *E*. *coli*, Table 3 lists the dominating combinations of STs and ESBL/AmpC genes for healthy and diseased animals. The most frequent ST in *E*. *coli* HA was ST101 (n = 6), followed by ST10 (n = 5). In *E*. *coli* DA, the top STs were ST10 (n = 5) and ST117/ST167 (n = 3 each). All ST10 isolates were retrieved across five countries from cattle, poultry, and swine and harbored four types of *bla*_ESBL_. All ST101 isolates harbored *bla*_CTX-M-1_ and were mainly isolated in healthy swine from Germany (n = 5). Four ST101 isolates retrieved from the swine from Germany (on the same branch in Fig 3A) had ≤ 1 gene of difference, whereas the two other ST101 isolated in Germany and Hungary had 40 and 42 genes of difference, respectively. *E*. *coli* ST354 (n = 4) were equally divided between healthy chickens from Hungary and diseased cattle from France, harboring *bla*_CMY-2_ and *bla*_CTX-M-15_, respectively. All three ST117 isolates found in DA from France, either from broilers (n = 2) or swine (n = 1), were harboring *bla*_CTX-M-1_ and *bla*_CTX-M-15_, respectively. All three ST167 isolates harbored *bla*_CTX-M-1_ and were retrieved from diseased cattle in France (n = 1) and Germany (n = 2). ST1011 were all isolated from HA broiler, raised in Spain (n = 2) or France and harbored either *bla*_CTX-M-14_ (n = 1) or *bla*_SHV-12_ (n = 2). The three ST88 isolates came from DA cattle, DA swine and HA swine raised in UK, France and Germany, respectively, and all produced a different ESBL/pAmpC. We retrieved only two *E*. *coli* ST131 isolates with *bla*_CTX-M-14_ and *bla*_CTX-M-15_ from a diseased swine in France and a diseased veal calf in Italy, respectively (Table 3).

**Table 3:** Dominating Sequence Types (ST) of *E*. *coli* producing ESBL/AmpC in healthy (HA) and diseased food-producing animals (DA). STs only represented by a single isolate in both HA and DA have been excluded from this overview.

	ST	Isolates (n)	Encoded ESBL/AmpC (n)
Healthy animals	101	6	CTX-M-1 (6)
	10	5	CTX-M-1 (2); SHV-12 (2); CTX-M-1/SHV-12 (1)
	1011	3	CTX-M-14 (1); SHV-12 (2)
	189	2	CTX-M-1 (1); TEM-52 (1)
	354	2	CMY-2 (2)
	770	2	CTX-M-14 (2)
Diseased animals	10	5	CTX-M-1 (2); CTX-M-14 (2); CTX-M-55 (1)
	117	3	CTX-M-1 (2); CTX-M-15 (1)
	167	3	CTX-M-1 (3)
	88	2	CTX-M-14 (1); CMY-2 (1)
	131	2	CTX-M-14 (1); CTX-M-15 (1)
	354	2	CTX-M-15 (1)
	617	2	CTX-M-15 (1); CTX-M-32 (1)
	1431	2	CTX-M-15 (2)

All *E*. *coli* isolates (78 out of 78; 100%) harbored one or several plasmids (1–9). Seventy-seven *E*. *coli* isolates harbored both ESBL- or pAmpC-encoding genes, and genes of plasmids which incompatibility groups are given in S4 Table. Thirty different Inc types were identified in different isolates and in different proportions. Most isolates (51 out of 78, 65.4%) harbored between 3 and 5 plasmids of different incompatibility groups. IncFII, IncFIB, and Incl1-Iγ were the most dominant Inc types detected (n = 63, 55, and 40, respectively).

*Salmonella* spp. HA clustered in seven STs, among which ST15 dominated (n = 17, 58.6%) followed by ST548 (n = 6, 20.7%). The phylogenetic trees revealed two clusters in Germany including the 17 isolates belonging to ST15 (serovar Heidelberg) and six isolates belonging to ST548 (serovar Minnesota) (Fig 3B). For the ST15 isolates, five patterns of resistance genes were observed (Fig 3B). All *Salmonella* spp. isolates (29 out of 29; 100%) harbored one or several plasmids (1–7). We retrieved 15 different incompatibility groups in *Salmonella* spp., with IncC (n = 22), colpVC (n = 19) and IncX1 (n = 17) dominating (S5 Table).

### Distribution of the virulence-associated genes

Although not the primary purpose of our study, virulence genes deserve some attention due to their role in the pathogenicity of animal-borne ESBL- and AmpC-producing Ec/Salm. Genome analysis retrieved a high load and diversity of virulence genes (S4 and S5 Tables). Virulence factors were found in all tested Ec/Salm isolates. We found 78 different virulence-associated genes in our *E*. *coli* genome collection. The majority of *E*. *coli* (n *=* 49; 62.8%) contained ≥ 10 virulence factors and 21 isolates (26.9%) harbored ≥ 20 virulence genes (S4 Table). The most prevalent genes were *terC* (n *=* 78; 100%), *traT* (n = 64; 82.1%), *gad* (n *=* 54; 69.2%), *sitA* (n = 48; 61.5%), *iss* (n = 47; 60.3%), and *ompT* (n = 45; 57.7%). One *E*. *coli*, isolated from a septicemia in cattle (ST8059, Italy) harbored a single virulence gene. Another *E*. *coli* isolated from cattle (ST6042; Italy) was recognized as Shiga toxin-producing *E*. *coli* (STEC). It carried *stx1A* and *stx1B* encoding Shiga toxin and *nleB/nleC* determining non-LEE encoded effectors (S4 Table).

We retrieved 124 different virulence-associated genes in our collection of *Salmonella* spp. genomes (S5 Table). All the isolates harbored ≥ 98 virulence genes. Six genes (*cdtB*, *gogB*, *sseI*, *sseK1*, *sspH1*, and *ybtS*) were rare and detected in ≤ eight *Salmonella* spp. isolates.

## Discussion

Our results are valuable data from two distinct populations of food-producing animals, namely healthy and diseased animals. To the best of our knowledge, it is the first report describing the ESBL/pAmpC carriage and the population structure of isolates collected from healthy animals at slaughterhouses (which will enter the food chain production) and from diseased food-producing animals in farms. Understanding the antimicrobial resistance spread among commensal and zoonotic isolates is of particular interest as it poses a threat to human health via the food chain [31]. Moreover, the comparison of resistance gene content between commensal/zoonotic and pathogenic isolates is of importance as it may help understanding the transmission of antimicrobial resistance between both populations [32, 33]. The resistance level to 3GC of *E*. *coli* and *Salmonella* spp. is in line with other reports [8, 13, 14, 34]. The overall prevalence of Ec/Salm producing ESBL/pAmpC was 2.0% (n = 107/5,331) and the various type of enzymes identified attested to their diversity, as previously reported [6, 21, 35].

It would also be relevant to determine the evolution of 3GC-R in livestock. For the HA population covered in the preceding EASSA projects [6, 36], the proportion of 3GC-R *E*. *coli* was 4.0% (109/2712) in 2008–09, 3.3% (100/2993) in 2013–2014, and 1.0% (34/3412) in the present study conducted in 2015–2018. Similar observations were made in *ad hoc* studies and in several European national surveys [14, 23, 24]. Hence, the French national surveillance network Resapath reported a decreasing proportion of ESBL/AmpC in food-producing animals in the time period 2011–2021 [37]. In addition, the Dutch national surveillance program MARAN found in 2019 that in the randomized, non-selective screening, for the first time in twenty years, no *E*. *coli* resistant to 3GCs were detected in fecal samples from broilers, pigs, dairy cattle and veal calves [23]. In 2019, a reduction in prevalence of animals carrying ESBL/AmpC-producing *E*. *coli* was observed in all livestock species compared to 2018, particularly in broilers [23]. This trend was confirmed in the most recent survey [38]. Since such decrease was observed in countries (France and The Netherlands) where antimicrobial usage has decreased considerably [37, 38], it is tempting to ascribe the decline of ESBL/AmpC prevalence to a reduction of the antibiotic consumption in livestock. For all EU countries, the European Surveillance of Veterinary Antimicrobial Consumption (ESVAC) is recording in a uniform manner national antimicrobial usage in veterinary medicine [25]. In the period 2011–2021, the overall consumption of antimicrobials in livestock has decreased over 47% for 25 countries providing sales data, with the consumption of 3GC falling by 38% in this period. Consequently, the decline of the prevalence of ESBL/AmpC in Europe is likely related to the decreased cephalosporin use [25]. A comparison of consumption per animal species would be very helpful in further elucidating this association, but unfortunately, such data are barely available to date. From 2024 onwards, it will become mandatory for EU countries to provide antimicrobial usage data by animal species under the framework of EU Regulation 2019/6.

The most frequent *bla* genes associated with the resistance to 3GC identified in Ec/Salm encoded CTX-M type enzymes (67.9%) and CMY-2 (75.9%), respectively (Figs 1 and 2). In addition, the nature of ESBL may also depend on the animal species and their geographic region. Hence, CTX-M-1 dominated in cattle from Germany and poultry from Germany, The Netherlands, and UK, whereas CTX-M-15 dominated in cattle from UK [4, 39]. Although rare in Europe, CTX-M-2 and CTX-M-8 were frequent in humans, companion animals, and cattle from South America [34, 40]. In Asia, CTX-M-14 was common in humans and in various animal groups such as pet animals and poultry [17]. In Africa, the few reports available indicated that CTX-M-15 and CTX-M-9 dominated in food-producing animals (livestock and poultry) and in humans [34, 41]. We retrieved the majority of ESBL/pAmpC producing Ec/Salm in broilers (51.4%) which was the animal species with the highest representation among HA isolates. The analysis of the genome collection retrieved a wide range of virulence-associated genes in HA and DA ESBL- and AmpC-producing *E*. *coli* and *Salmonella* spp. from European livestock, and deserves further research.

3GC-R *E*. *coli* isolates were more prevalent in DA (5.4%) compared with HA (1.4%). These results were consistent with those observed in Europe for healthy and diseased food-producing animals [6, 20]. However, higher prevalence (29% to 57%) has been observed in geographical areas outside Europe (*e*.*g*. healthy poultry from Ghana, Nepal, or China) [41–43]. Overall, CTX-M-1, SHV-12, and CMY-2 (34.6%, 12.8%, and 14.1%) were the most common ESBLs and pAmpC identified in the whole collection of 3CG-R *E*. *coli*, independent of the animal source and species. Then, we frequently identified *bla*_CTX-M-14_ and *bla*_CTX-M-15_ (11.5% for each) among the 3GC-R *E*. *coli* collection. More specifically, this ranking differed between HA and DA reservoirs, independent of the animal species. We found all the same ranking for the HA reservoir, with CTX-M-1 as the most identified (n = 13, 38.0%) followed by both SHV-12 and CMY-2 (n = 7, 21.0% each). Regarding the DA reservoir, although CTX-M-1 (n = 14, 32%) dominated, CMY-2 and SHV-12 were in fourth and fifth rank (n = 4, 9% and n = 3, 7%, respectively), surpassed by CTX-M-15 (n = 7, 16%) and CTX-M-14 (n = 6, 14%). We confirmed previous European data findings showing that CTX-M-1, SHV12, and CMY-2 dominated in HA, whereas CTX-M-1, CTX-M-14, and CTX-M-15 dominated mainly in DA [20, 35]. In low-income areas, *bla*_CTX-M-15_ reached a higher prevalence in poultry, *i*.*e*. 30.1% in Nepal and 96.0% in Ghana [41, 42].

The large genetic diversity of the 3GC-R *E*. *coli* population (n = 78) was illustrated by the 51 STs identified, of which 68.6% were represented by a single isolate (S1 Table and Fig 3A). We found major international high-risk clones including ST10 (12.8%), ST354 (5.1%), ST131 (2.6%) and ST410 (1.3%), while others (ST405, ST648, ST38, ST73, and ST1193) were absent from the present collections. The dominance of ST10 has already been described in DA, in livestock, and in humans [44]. We found only two isolates of the epidemic extra-intestinal pathogenic ST131 originating from unrelated DA (*i*.*e*. raised in different countries). One of the isolates harbored CTX-M-14 and the other CTX-M-15 as described in humans and to a lesser extent in animals [6, 7, 17]. No *E*. *coli* cluster was identified, either within a reservoir (HA or DA) or between reservoirs (Fig 3A). We only highlighted the spread of *E*. *coli* ST101 producing CTX-M-1 in healthy swine from Germany (n = 5, see the green sector in Fig 3A). The five *E*. *coli* ST101 isolates were recovered in 2017 (one abattoir resulting in 1 *E*. *coli* ST101) and in 2018 from different swine (another abattoir with 4 *E*. *coli* ST101 from swine with body weights 93, 109, 103, and 89 kg), and according to our protocol one sample per farm was collected. However, for privacy reasons the abattoirs are unable to provide the location of the farms. Addition plasmid research might unravel this cluster of isolates. Overall, *E*. *coli* isolates producing ESBL/pAmpC were disseminated in HA or DA by many STs of *E*. *coli* (Fig 3A), ruling out the dispersal of 3GC-R via a single carrier clone and underlining the limited clone transfers between food-producing animals.

The prevalence of *Salmonella* in animals is very low, which has caused differences in the size of the 3GC-R *Salmonella* spp. collections retrieved from HA (n = 28) and DA (n = 1) and preventing their comparison. Moreover, the clonality of *Salmonella* may be biased, as the majority of isolates were obtained from broilers in Germany. In total, *bla*_CMY-2_ was the most frequent resistant determinant to 3GC, confirming other reports [14, 45, 46]. We identified two clusters with serovars Heidelberg (ST15; 15 isolates) and Minnesota (ST548; 6 isolates) harboring *bla*_CMY-2_ in healthy German poultry. *S*. Heidelberg isolates were recovered from samples of two slaughter sites collected on different days evenly spread in the period of December 2016 to September 2017. *S*. Minnesota isolates were also recovered at two slaughter sites, on different sampling days during October 2016 to May 2017. The locations of the farms are not available to us, but it is has been confirmed and warranted by the provider that all *S*. Heidelberg and *S*. Minnesota isolates but one originate from different farms. It is of interest that in a follow–up study in 2021–2022 249 *Salmonella* isolates have been collected and serotyped in Germany, but none of them belonged to the aforementioned two serovars. A sub-analysis of SNPs might shed light on the presence or absence of clonal relationships. Another explanation could be a contamination of *Salmonella* during the production chain, i.e., breeder farm, production farm, transportation, abattoir and retail. The slaughterhouses and also the transport cages have been identified as potential sources of *Salmonella* contamination of poultry meat in various studies [47]. During slaughter, carcasses may become contaminated by *Salmonella* found in the intestinal content of the birds, either from within the flock or in previously slaughtered flocks [47, 48]. Interestingly, two *bla*_CMY-2_ negative isolates of the epidemic ST15 *S*. Heidelberg harbored *bla*_SHV-12_ or *bla*_CTX-M-8_ (Fig 3B). In the USA, *S*. Heidelberg has frequently been implicated in human infections related to consumption of pork and chicken meat [13, 14, 49]. Moreover, *S*. Heidelberg producing CMY-2 was frequently isolated from chicken and swine from North and South America [13, 14, 50, 51]. In contrast, *S*. Minnesota producing CMY-2 was rarer but already isolated from poultry meat produced in Brazil [45, 52, 53].

This study has some limitations. Although we tested 5,331 Enterobacterales across up to twelve European countries in three animal species, we retrieved only 107 3GC-R isolates with an imbalance in the size of the collection from HA and DA (especially for *Salmonella* spp.). The distribution of 3GC-R isolates between countries, animal species and healthy or diseased status introduces a level of bias, as the resulting collection is not balanced between countries and animal species (Table 1). The representation of each country was partly uneven, preventing further comparison. Another limitation of the study is the lack of complete demographic data in Germany, which precludes definitive conclusions on clonal relationships. Nevertheless, the *E*. *coli* isolates included originated from several countries and their whole-genome sequencing associated with cgMLST allowed a complete population structure description. However, the low prevalence of *Salmonella* spp. in animals precludes strong conclusions.

## Conclusions

The prevalence of 3GC-resistant *E*. *coli* and *Salmonella* spp. was low in HA (1.4%) and DA (5.4%). *bla*_CTX-M-1_ dominated in a highly diverse population of *E*. *coli*. No clusters have been identified between *E*. *coli* isolated from HA and DA, with high *E*. *coli* clonal diversity suggesting multiple origins of contamination. This contrasted with the clonal population of 3GC-R *Salmonella* spp. in which *bla*_CMY-2_ dominated through two major serovars.

## Supporting information

S1 Table ESBL/pAmpC distribution according to animal, country, and origin (Healthy Animals, HA; Diseased Animals, DA) of 78 *E. coli* isolates retrieved in Europe between 2015 and 2018. (DOCX)

S2 Table ESBL/pAmpC distribution according to animal, country, and origin (Healthy Animals, HA; Diseased Animals, DA) of *29 Salmonella* spp. isolates retrieved in Europe between 2015 and 2018. (DOCX)

S3 Table Multidrug resistant *E*. *coli* and *Salmonella* spp. isolates producing ESBL and pAmpC in healthy animals in Europe between 2015 and 2018. AMP, ampicillin; AZM, Azithromycin; FEP, Cefepime ; CTX, cefotaxime; CAZ ceftazidime; CHL, chloramphenicol; CIP, ciprofloxacin; CT, colistin; GEN, gentamicin; MEM, Meropenem; NA, nalidixic acid; TMP, trimethoprim ; SMZ, sulfamethoxazole; TET, tetracycline; TGC, Tigecycline; SXT trimethoprim/sulfamethoxazole.
(DOCX)

S4 Table Line listings of *E*. *coli* strains. (XLSX)

S5 Table Line listings of *Salmonella* spp. strains. (XLSX)
