# Supplementary material for: Genome analysis of third-generation cephalosporin-resistant Escherichia coli and Salmonella species recovered from healthy and diseased food-producing animals in Europe
Source: PLoS One. 2023 Oct 26;18(10):e0289829. doi: 10.1371/journal.pone.0289829 (PMC10602299; doi:10.1371/journal.pone.0289829)
Supplement: S1 Table — (DOCX) [file pone.0289829.s001.docx]

|  | **ESBL/AmpC** | | | | **ST (number of isolates)** | **Country (number of isolates)** | | | | | | | | | | | | | | | |
| --- | --- | --- | --- | --- | --- | --- | --- | --- | --- | --- | --- | --- | --- | --- | --- | --- | --- | --- | --- | --- | --- |
|  | **Type** |  | **No. of isolates** | |  | **Belgium** | | **France** | | **Germany** | | **Hungary** | | **Italy** | | **The Netherlands** | | **Spain** | | **United Kingdom** | |
|  |  |  | **N** | **%** |  | **HA (0)** | **DA (3)** | **HA (5)** | **DA**  **(24)** | **HA (7)** | **DA**  **(4)** | **HA (9)** | **DA (0)** | **HA (2)** | **DA (8)** | **HA (2)** | **DA (0)** | **HA (8)** | **DA (0)** | **HA (1)** | **DA (5)** |
| TOTAL (N=78) | CTX-M-1 | | 27 | 34.6 | ST10 (4), ST88 (1), ST101 (6), ST117 (2), ST167 (3), ST189 (1), ST 227 (1), ST 359 (1), ST448 (1), ST609 (1), ST844 (1), ST1286 (1), ST 2008 (1), ST4247 (1), ST6042 (1), ST8059 (1) | 0 | 1 | 2 | 8 | 7 | 4 | 2 | 0 | 1 | 1 | 1 | 0 |  |  |  |  |
|  | CTX-M-14 | | 9 | 11.5 | ST10 (2), ST88 (1), ST130 (1), ST131 (1), ST770 (2), ST1011 (1), new ST* (1) |  |  | 0 | 3 |  |  |  |  |  |  |  |  | 3 | 0 | 0 | 3 |
|  | CTX-M-15 | | 9 | 11.5 | ST90 (1), ST117 (1), ST131 (1), ST354 (2), ST617 (1), ST1431 (2) ST4252 (1) |  |  | 1 | 3 |  |  |  |  | 1 | 3 |  |  |  |  | 0 | 1 |
|  | CTX-M-32 | | 2 | 2.5 | ST617 (1), ST2137 (1) |  |  | 0 | 1 |  |  |  |  | 0 | 1 |  |  |  |  |  |  |
|  | CTX-M-55 | | 3 | 3.9 | ST10 (1), ST56 (1), ST1340 (1) |  |  | 0 | 2 |  |  |  |  |  |  |  |  |  |  | 0 | 1 |
|  | SHV-12 | | 10 | 12.8 | ST10 (2), ST40 (1), ST57 (1), ST410 (1), ST710 (1), ST1011 (2), ST1308 (1), ST4980 (1) |  |  | 2 | 2 |  |  |  |  | 0 | 1 | 1 | 0 | 4 | 0 |  |  |
|  | TEM-52 | | 3 | 3.9 | ST189 (1), ST772 (1), ST877 (1) |  |  | 0 | 2 |  |  |  |  |  |  |  |  | 1 | 0 |  |  |
|  | CMY-2 | | 11 | 14.1 | ST23 (1), ST88 (1), ST117 (1), ST140 (1,), ST354 (2), ST429 (1), ST641 (1), ST1196 (1), ST1594 (1), ST2223 (1) | 0 | 2 | 0 | 1 |  |  | 7 | 0 | 0 | 1 |  |  |  |  |  |  |
|  | SHV-12 and CTX-M-1 | | 1 | 1.3 | ST10 (1) |  |  |  |  |  |  |  |  |  |  |  |  |  |  | 1 | 0 |
|  | CTX-M-1 and CTX-M-14 | | 1 | 1.3 | ST224 (1) |  |  | 0 | 1 |  |  |  |  |  |  |  |  |  |  |  |  |
|  | CMY-2 and CTX-M-15 | | 1 | 1.3 | ST162 (1) |  |  |  |  |  |  |  |  | 0 | 1 |  |  |  |  |  |  |
|  | None ESBL/AmpC | | 1 | 1.3 | ST783 (1) |  |  | 0 | 1 |  |  |  |  |  |  |  |  |  |  |  |  |
| CATTLE (N=25) | CTX-M-1 | | 9 | 36.0 | ST10 (1), ST167 (3), ST227 (1), ST448 (1), ST844 (1), ST6042 (1), ST8059 (1) |  |  | 0 | 3 | 0 | 4 |  |  | 1 | 1 |  |  |  |  |  |  |
|  | CTX-M-14 | | 4 | 16.0 | ST10 (1) , ST88(1), ST130 (1), new ST* (1) |  |  | 0 | 1 |  |  |  |  |  |  |  |  |  |  | 0 | 3 |
|  | CTX-M-15 | | 6 | 24.0 | ST131 (1), ST354 (2), ST1431 (2) ST4252 (1) |  |  | 0 | 2 |  |  |  |  | 1 | 3 |  |  |  |  |  |  |
|  | CTX-M-32 | | 1 | 4.0 | ST2137 (1) |  |  |  |  |  |  |  |  | 0 | 1 |  |  |  |  |  |  |
|  | CTX-M-55 | | 1 | 4.0 | ST56 (1) |  |  |  |  |  |  |  |  |  |  |  |  |  |  | 0 | 1 |
|  | SHV-12 | | 1 | 4.0 | ST710 (1) |  |  |  |  |  |  |  |  | 0 | 1 |  |  |  |  |  |  |
|  | CMY-2 | | 1 | 4.0 | ST641 (1) |  |  |  |  |  |  |  |  | 0 | 1 |  |  |  |  |  |  |
|  | CMY-2 and CTX-M-15 | | 1 | 4.0 | ST162 (1) |  |  |  |  |  |  |  |  | 0 | 1 |  |  |  |  |  |  |
|  | None ESBL/AmpC | | 1 | 4.0 | ST783 (1) |  |  | 0 | 1 |  |  |  |  |  |  |  |  |  |  |  |  |
| POULTRY (N=27) | CTX-M-1 | | 7 | 25.9 | ST10 (1), ST101 (1), ST117 (2), ST189 (1), ST1286 (1), ST2008 (1) |  |  | 2 | 2 |  |  | 2 | 0 |  |  | 1 | 0 |  |  |  |  |
|  | CTX-M-14 | | 3 | 11.1 | ST770 (2), ST1011 (1) |  |  |  |  |  |  |  |  |  |  |  |  | 3 | 0 |  |  |
|  | SHV-12 | | 6 | 22.2 | ST10 (1), ST40 (1), ST57 (1), ST1011 (2), ST4980 (1) |  |  | 2 | 0 |  |  |  |  |  |  | 1 | 0 | 3 | 0 |  |  |
|  | TEM-52 | | 1 | 3.7 | ST189 (1) |  |  |  |  |  |  |  |  |  |  |  |  | 1 | 0 |  |  |
|  | CMY-2 | | 9 | 33.4 | ST23 (1), ST117 (1), ST140 (1,), ST354 (2), ST429 (1), ST1196 (1), ST1594 (1), ST2223 (1) | 0 | 2 |  |  |  |  | 7 | 0 |  |  |  |  |  |  |  |  |
|  | SHV-12 and CTX-M-1 | | 1 | 3.7 | ST10 (1) |  |  |  |  |  |  |  |  |  |  |  |  |  |  | 1 | 0 |
| PIG (N=26) | CTX-M-1 | | 11 | 42.3 | ST10 (2), ST88 (1), ST101 (5), ST359 (1), ST609 (1), ST4247 (1) | 0 | 1 | 0 | 3 | 7 | 0 |  |  |  |  |  |  |  |  |  |  |
|  | CTX-M-14 | | 2 | 7.7 | ST10 (1), ST131 (1) |  |  | 0 | 2 |  |  |  |  |  |  |  |  |  |  |  |  |
|  | CTX-M-15 | | 3 | 11.6 | ST90 (1), ST117 (1), ST617 (1) |  |  | 1 | 1 |  |  |  |  |  |  |  |  |  |  | 0 | 1 |
|  | CTX-M-32 | | 1 | 3.8 | ST617 (1), |  |  | 0 | 1 |  |  |  |  |  |  |  |  |  |  |  |  |
|  | CTX-M-55 | | 2 | 7.7 | ST10 (1), ST1340 (1) |  |  | 0 | 2 |  |  |  |  |  |  |  |  |  |  |  |  |
|  | SHV-12 | | 3 | 11.6 | ST10 (1), ST410 (1), ST1308 (1) |  |  | 0 | 2 |  |  |  |  |  |  |  |  | 1 | 0 |  |  |
|  | TEM-52 | | 2 | 7.7 | ST772 (1), ST877 (1) |  |  | 0 | 2 |  |  |  |  |  |  |  |  |  |  |  |  |
|  | CMY-2 | | 1 | 3.8 | ST88 (1) |  |  | 0 | 1 |  |  |  |  |  |  |  |  |  |  |  |  |
|  | CTX-M-1 and CTX-M-14 | | 1 | 3.8 | ST224 (1) |  |  | 0 | 1 |  |  |  |  |  |  |  |  |  |  |  |  |

**Supplementary Table S1. ESBL/pAmpC distribution according to animal, country, and origin (Healthy Animals, HA; Diseased Animals, DA) of 78 *E. coli* isolates retrieved in Europe between 2015 and 2018*.***

* The allele combination of the new ST was: *adk* (new), *fumC* (7), *gyrB* (1), *icd* (1), *mdh* (8), *purA* (18), *recA* (6)
