# Supplementary material for: Genome analysis of third-generation cephalosporin-resistant Escherichia coli and Salmonella species recovered from healthy and diseased food-producing animals in Europe
Source: PLoS One. 2023 Oct 26;18(10):e0289829. doi: 10.1371/journal.pone.0289829 (PMC10602299; doi:10.1371/journal.pone.0289829)
Supplement: S2 Table — (DOCX) [file pone.0289829.s002.docx]

|  | **ESBL/AmpC** | | | **Serovars** | **ST (number of isolates)** | **Country (number of isolates)** | | | | | |
| --- | --- | --- | --- | --- | --- | --- | --- | --- | --- | --- | --- |
|  | **Type** | **No. of isolates** | |  |  | **France** | | **Germany** | | **Hungary** | |
|  |  | **N** | **%** |  |  | **HA (0)** | **DA (1)** | **HA (26)** | **DA (0)** | **HA (2)** | **DA (0)** |
| **POULTRY (N=28)** | CTX-M-3 | 1 | 3.57 | Infantis (1) | New ST* (1) |  |  | 1 |  |  |  |
|  | CTX-M-8 | 1 | 3.57 | Heidelberg (1) | ST15 (1) |  |  | 1 |  |  |  |
|  | CTX-M-14 | 2 | 7.15 | Infantis (2) | ST32 (2) |  |  |  |  | 2 |  |
|  | SHV-12 | 1 | 3.57 | Heidelberg (1) | ST15 (1) |  |  | 1 |  |  |  |
|  | TEM-52 | 1 | 3.57 | Derby (1) | ST3871 (1) |  |  | 1 |  |  |  |
|  | CMY-2 | 21 | 75.00 | Heidelberg (14)  Saint Paul (1)  Minnesota (6) | ST15 (14)  ST50 (1)  ST548 (6) |  |  | 21 |  |  |  |
|  | CMY-2 and CTX-M-2 | 1 | 3.57 | Heidelberg (1) | ST15 (1) |  |  | 1 |  |  |  |
| **CATTLE (N=1)** | CTX-M-55 | 1 | 100.00 | Unknown | ST34 (1) |  | 1 |  |  |  |  |

**Supplementary Table S2. ESBL/pAmpC distribution according to animal, country, and origin (Healthy Animals, HA; Diseased Animals, DA) of *29 Salmonella* spp. isolates retrieved in Europe between 2015 and 2018*.***

* The allele combination of the new ST was: *aroC* (17), *dnaN* (new), *hemD* (22), *hisD* (17), *purE* (5), *sucA* (21), *thrA* (19).
