# Supplementary material for: Genome analysis of third-generation cephalosporin-resistant Escherichia coli and Salmonella species recovered from healthy and diseased food-producing animals in Europe
Source: PLoS One. 2023 Oct 26;18(10):e0289829. doi: 10.1371/journal.pone.0289829 (PMC10602299; doi:10.1371/journal.pone.0289829)
Supplement: S3 Table — AMP, ampicillin; AZM, Azithromycin; FEP, Cefepime ; CTX, cefotaxime; CAZ ceftazidime; CHL, chloramphenicol; CIP, ciprofloxacin; CT, colistin; GEN, gentamicin; MEM, Meropenem; NA, nalidixic acid; TMP, trimethoprim ; SMZ, sulfamethoxazole; TET, tetracycline; TGC, Tigecycline; SXT trimethoprim/sulfamethoxazole. (DOCX) [file pone.0289829.s003.docx]

**Supplementary Table S3. Multidrug resistant *E. coli* and *Salmonella* spp. isolates producing ESBL and pAmpC in healthy animals in Europe between 2015 and 2018.** AMP, ampicillin; AZM, Azithromycin; FEP, Cefepime ; CTX, cefotaxime; CAZ ceftazidime; CHL, chloramphenicol; CIP, ciprofloxacin; CT, colistin; GEN, gentamicin; MEM, Meropenem; NA, nalidixic acid; TMP, trimethoprim ; SMZ, sulfamethoxazole; TET, tetracycline; TGC, Tigecycline; SXT trimethoprim/sulfamethoxazole.

|  |  |  |  | **CMI (mg/L) (Interpretation according to EUCAST breakpoints)** | | | | | | | | | | | | | | | |
| --- | --- | --- | --- | --- | --- | --- | --- | --- | --- | --- | --- | --- | --- | --- | --- | --- | --- | --- | --- |
| **Isolate** | **Pathogen** | **Animal** | **Country** | **AMP** | **AZM** | **FEP** | **CTX** | **CAZ** | **CHL** | **CIP** | **CT** | **GEN** | **MEM** | **NA** | **TMP** | **SMZ** | **TET** | **TGC** | **SXT** |
| EASSA-1 | *E. coli* | Cattle | Italy | > 256 (R) | 16 (S) | 8 (I) | > 64 (R) | 2 (S) | > 256 (R) | 0.25 (S) | 0.5 (S) | 0.5 (S) | ≤ 0.015 (S) | 16 (S) | > 512 (R) | 16 (S) | 2 (S) | 0.25 (S) | > 256 (R) |
| EASSA-2 | *E. coli* | Cattle | Italy | > 256 (R) | 4 (S) | 4 (I) | > 64 (R) | 8 (I) | 8 (S) | 0.5 (I) | 0.25 (S) | 0.5 (S) | ≤ 0.015 (S) | 16 (S) | > 512 (R) | > 1024 (R) | 64 (R) | 0.5 (S) | 256 (R) |
| EASSA-3 | *E. coli* | Chicken | The Netherlands | > 256 (R) | 4 (S) | 4 (I) | 64 (R) | 2 (S) | 8 (S) | 0.008 (S) | 0.25 (S) | 1 (S) | ≤ 0.015 (S) | 4 (S) | 0.12 (S) | > 1024 (R) | 128 (R) | 0.5 (S) | 0.25 (S) |
| EASSA-30 | *E. coli* | Pig | Germany | > 256 (R) | 2 (S) | 32 (R) | > 64 (R) | 4 (S) | 8 (S) | 0.25 (S) | 0.5 (S) | 1 (S) | ≤ 0.015 (S) | > 128 (R) | > 512 (R) | > 1024 (R) | 64 (R) | 0.5 (S) | > 256 (R) |
| EASSA-31 | *E. coli* | Pig | Germany | > 256 (R) | 8 (S) | 16 (R) | > 64 (R) | 4 (S) | 8 (S) | 4 (R) | 0.25 (S) | 2 (S) | ≤ 0.015 (S) | > 128 (R) | 0.5 (S) | 32 (S) | 128(R) | 0.25 (S) | 0.06(S) |
| EASSA-33 | *E. coli* | Chicken | Spain | > 256 (R) | 4 (S) | 2 (S) | 64 (R) | 1 (S) | 4 (S) | 4 (R) | 0.25 (S) | 64 (R) | ≤ 0.015 (S) | > 128 (R) | 0.12 (S) | 1024 (R) | 32 (R) | 0.5 (S) | 0.06 (S) |
| EASSA-34 | *E. coli* | Chicken | Spain | > 256 (R) | 4 (S) | 4 (I) | > 64 (R) | 1 (S) | 4 (S) | 4 (R) | 0.25 (S) | 64 (R) | 0.03 (S) | > 128 (R) | 0.25 (S) | > 1024 (R) | 32 (R) | 1 (S) | 0.06 (S) |
| EASSA-35 | *E. coli* | Chicken | Spain | > 256 (R) | 4 (S) | 0.5 (S) | 4 (R) | 16 (R) | 8 (S) | 8 (R) | 0.25 (S) | 1 (S) | ≤ 0.015 (S) | > 128 (R) | > 512 (R) | > 1024 (R) | 128 (R) | 0.5 (S) | > 256 (R) |
| EASSA-36 | *E. coli* | Pig | France | > 256 (R) | 4 (S) | 0.25 (S) | 32 (R) | 8 (I) | 128 (R) | 16 (R) | 0.25 (S) | 64 (R) | ≤ 0.015 (S) | > 128 (R) | 0.25 (S) | > 1024 (R) | 64 (R) | 0.25 (S) | 0.03 (S) |
| EASSA-43 | *E. coli* | Chicken | France | > 256 (R) | 4 (S) | 4 (I) | > 64 (R) | 1 (S) | 8 (S) | 0.12 (S) | 0.25 (S) | 1 (S) | ≤ 0.015 (S) | 128 (R) | > 512 (R) | > 1024 (R) | 2 (S) | 0.25 (S) | > 256 (R) |
| EASSA-46 | *E. coli* | Chicken | France | > 256 (R) | 4 (S) | 0.5 (S) | 4 (R) | 64 (R) | 8 (S) | 8 (R) | 0.5 (S) | 2 (S) | ≤ 0.015 (S) | > 128 (R) | > 512 (R) | > 1024 (R) | 128 (R) | 0.5 (S) | > 256 (R) |
| EASSA-53 | *E. coli* | Chicken | The Netherlands | > 256 (R) | 4 (S) | 0.25 (S) | 2 (I) | 16 (R) | 128 (R) | 0.008 (S) | 0.5 (S) | 1 (S) | ≤ 0.015 (S) | 4 (S) | 0.25 (S) | > 1024 (R) | 2 (S) | 0.5 (S) | 0.25 (S) |
| EASSA-60 | *E. coli* | Chicken | Hungary | > 256 (R) | 4 (S) | 0.25 (S) | 8 (R) | 16 (R) | 8 (S) | 8 (R) | 0.25 (S) | 256 (R) | 0.03 (S) | > 128 (R) | > 512 (R) | > 1024 (R) | 256 (R) | 0.5 (S) | > 256 (R) |
| EASSA-61 | *E. coli* | Chicken | Hungary | > 256 (R) | 4 (S) | 0.25 (S) | 8 (R) | 16 (R) | 8 (S) | 8 (R) | 0.25 (S) | 256 (R) | 0.03 (S) | > 128 (R) | > 512 (R) | > 1024 (R) | 256 (R) | 0.25 (S) | > 256 (R) |
| EASSA-62 | *E. coli* | Chicken | Hungary | 256 (R) | 4 (S) | 0.25 (S) | 8 (R) | 16 (R) | 8 (S) | 0.25 (S) | 0.25 (S) | 1 (S) | ≤ 0.015 (S) | 128 (R) | 0.12 (S) | 32 (S) | 2 (S) | 0.5 (S) | 0.03 (S) |
| EASSA-63 | *E. coli* | Chicken | Hungary | > 256 (R) | 8 (S) | 4 (I) | 64 (R) | 1 (S) | 8 (S) | 0.12 (S) | 0.5 (S) | 1 (S) | ≤ 0.015 (S) | 64 (R) | 0.12 (S) | > 1024 (R) | 2 (S) | 1 (S) | 0.25 (S) |
| EASSA-64 | *E. coli* | Chicken | Hungary | 256 (R) | 4 (S) | 0.12 (S) | 8 (R) | 16 (R) | 8 (S) | 8 (R) | 0.25 (S) | 2 (S) | ≤ 0.015 (S) | > 128 (R) | > 512 (R) | > 1024 (R) | 128 (R) | 0.25 (S) | > 256 (R) |
| EASSA-65 | *E. coli* | Chicken | Hungary | 256 (R) | 4 (S) | 0.12 (S) | 8 (R) | 16 (R) | 8 (S) | 32 (R) | 0.25 (S) | 1 (S) | ≤ 0.015 (S) | > 128 (R) | 256 (R) | 128 (S) | 4 (S) | 0.25 (S) | 0.03 (S) |
| EASSA-66 | *E. coli* | Chicken | Hungary | 256 (R) | 4 (S) | 0.12 (S) | 4 (R) | 4 (S) | 64 (R) | 0.06 (S) | 0.25 (S) | 64 (R) | ≤ 0.015 (S) | 32 (R) | 0.12 (S) | > 1024 (R) | 64 (R) | 0.25 (S) | 0.12 (S) |
| EASSA-67 | *E. coli* | Chicken | Hungary | 256 (R) | 2 (S) | 0.25 (S) | 8 (R) | 16 (R) | 8 (S) | 8 (R) | 0.25 (S) | 1 (S) | 0.03 (S) | > 128 (R) | 0.5 (S) | 16 (S) | 2 (S) | 0.25 (S) | 0.03 (S) |
| EASSA-68 | *E. coli* | Chicken | Hungary | > 256 (R) | 8 (S) | 8 (I) | 64 (R) | 4 (S) | 16 (I) | 0.03 (S) | 0.5 (S) | 1 (S) | 0.03 (S) | 8 (S) | 0.5 (S) | > 1024 (R) | 32 (R) | 0.5 (S) | 0.5 (S) |
| EASSA-69 | *E. coli* | Pig | Germany | > 256 (R) | 4 (S) | 2 (S) | 32 (R) | 1 (S) | 8 (S) | 0.008 (S) | 0.25 (S) | 1 (S) | ≤ 0.015 (S) | 2 (S) | > 512 (R) | > 1024 (R) | 2 (S) | 0.25 (S) | > 256 (R) |
| EASSA-70 | *E. coli* | Pig | Germany | > 256 (R) | 4 (S) | 2 (S) | 16 (R) | 1 (S) | 8 (S) | 0.008 (S) | 0.12 (S) | 1 (S) | ≤ 0.015 (S) | 2 (S) | > 512 (R) | > 1024 (R) | 2 (S) | 0.25 (S) | > 256 (R) |
| EASSA-71 | *E. coli* | Pig | Germany | > 256 (R) | 4 (S) | 4 (I) | 32 (R) | 1 (S) | 8 (S) | 0.008 (S) | 0.25 (S) | 1 (S) | ≤ 0.015 (S) | 2 (S) | > 512 (R) | > 1024 (R) | 2 (S) | 0.25 (S) | > 256 (R) |
| EASSA-72 | *E. coli* | Pig | Germany | > 256 (R) | 4 (S) | 2 (S) | 32 (R) | 1 (S) | 8 (S) | 0.008 (S) | 0.25 (S) | 1 (S) | ≤ 0.015 (S) | 2 (S) | 0.25 (S) | 16 (S) | 2 (S) | 0.25 (S) | 0.06 (S) |
| EASSA-73 | *E. coli* | Pig | Germany | > 256 (R) | 4 (S) | 2 (S) | 16 (R) | 1 (S) | 8 (S) | 0.008 (S) | 0.12 (S) | 1 (S) | ≤ 0.015 (S) | 2 (S) | > 512 (R) | > 1024 (R) | 2 (S) | 0.25 (S) | > 256 (R) |
| EASSA-74 | *E. coli* | Chicken | United Kingdom | > 256 (R) | 4 (S) | 0.25 (S) | 2 (I) | 16 (R) | 32 (R) | 0.015 (S) | 0.25 (S) | 1 (S) | ≤ 0.015 (S) | 2 (S) | > 512 (R) | > 1024 (R) | 64 (R) | 0.5 (S) | > 256 (R) |
| EASSA-75 | *E. coli* | Chicken | France | > 256 (R) | 4 (S) | 4 (I) | 32 (R) | 1 (S) | 8 (S) | 0.12 (S) | 0.25 (S) | 2 (S) | ≤ 0.015 (S) | 128 (R) | 0.25 (S) | 16 (S) | 2 (S) | 0.25 (S) | 0.03 (S) |
| EASSA-76 | *E. coli* | Chicken | France | > 256 (R) | 4 (S) | 0.12 (S) | 1 (S) | 8 (I) | 32 (R) | 0.015 (S) | 0.25 (S) | 2 (S) | ≤ 0.015 (S) | 4 (S) | > 512 (R) | > 1024 (R) | 4 (S) | 0.25 (S) | > 256 (R) |
| EASSA-77 | *E. coli* | Pig | Spain | > 256 (R) | 4 (S) | 1 (S) | 16 (R) | 32 (R) | > 256 (R) | 0.12 (S) | 0.5 (S) | 1 (S) | ≤ 0.015 (S) | 128 (R) | > 512 (R) | 32 (S) | 32 (R) | 0.25 (S) | 2 (S) |
| EASSA-78 | *E. coli* | Chicken | Spain | > 256 (R) | 16 (S) | 4 (I) | 32 (R) | 1 (S) | 8 (S) | 8 (R) | 0.5 (S) | 128 (R) | ≤ 0.015 (S) | > 128 (R) | > 512 (R) | > 1024 (R) | 64 (R) | 0.5 (S) | > 256 (R) |
| EASSA-79 | *E. coli* | Chicken | Spain | > 256 (R) | 8 (S) | 0.5 (S) | 2 (I) | 16 (R) | 64 (R) | 8 (R) | 0.5 (S) | 1 (S) | ≤ 0.015 (S) | > 128 (R) | > 512 (R) | > 1024 (R) | 64 (R) | 0.25 (S) | > 256 (R) |
| EASSA-80 | *E. coli* | Chicken | Spain | > 256 (R) | 8 (S) | 0.5 (S) | 4 (R) | 4 (S) | 8 (S) | 8 (R) | 0.25 (S) | 2 (S) | ≤ 0.015 (S) | > 128 (R) | > 512 (R) | > 1024 (R) | 256 (R) | 0.25 (S) | > 256 (R) |
| EASSA-81 | *E. coli* | Chicken | Spain | > 256 (R) | 8 (S) | 0.5 (S) | 2 (I) | 16 (R) | 8 (S) | 1 (R) | 0.25 (S) | 1 (S) | ≤ 0.015 (S) | > 128 (R) | 0.25 (S) | 16 (S) | 4 (S) | 0.25 (S) | 0.06 (S) |
| EASSA-4 | *Salmonella spp.* | Chicken | Germany | 128 (R) | 4 (S) | 0.25 (S) | 8 (R) | 16 (R) | 4 (S) | 0.008 (S) | 0.5 (S) | 1 (S) | 0.03 (S) | 4 (S) | 0.25 (S) | > 1024 (R) | 128 (R) | 1 (S) | 0.25 (S) |
| EASSA-5 | *Salmonella spp.* | Chicken | Germany | 256 (R) | 8 (S) | 0.25 (S) | 8 (R) | 32 (R) | 8 (S) | 0.25 (I) | 0.5 (S) | 1 (S) | 0.03 (S) | > 128 (R) | 0.12 (S) | > 1024 (R) | 128 (R) | 2 (R) | 0.25 (S) |
| EASSA-6 | *Salmonella spp.* | Chicken | Germany | 256 (R) | 4 (S) | 0.25 (S) | 8 (R) | 32 (R) | 8 (S) | 0.25 (I) | 0.25 (S) | 1 (S) | 0.03 (S) | > 128 (R) | 0.12 (S) | > 1024 (R) | 128 (R) | 2 (R) | 0.25 (S) |
| EASSA-7 | *Salmonella spp.* | Chicken | Germany | 256 (R) | 8 (S) | 0.25 (S) | 8 (R) | 32 (R) | 8 (S) | 0.25 (I) | 0.5 (S) | 1 (S) | 0.03 (S) | > 128 (R) | 0.12 (S) | > 1024 (R) | 128 (R) | 2 (R) | 0.25 (S) |
| EASSA-8 | *Salmonella spp.* | Chicken | Germany | 256 (R) | 4 (S) | 0.25 (S) | 8 (R) | 32 (R) | 8 (S) | 0.25 (I) | 0.25 (S) | 1 (S) | 0.03 (S) | > 128 (R) | 0.12 (S) | > 1024 (R) | 128 (R) | 2 (R) | 0.25 (S) |
| EASSA-9 | *Salmonella spp.* | Chicken | Germany | 128 (R) | 4 (S) | 0.25 (S) | 8 (R) | 32 (R) | 64 (R) | 0.12 (I) | 0.25 (S) | 1 (S) | 0.03 (S) | > 128 (R) | 0.25 (S) | > 1024 (R) | 128 (R) | 1 (S) | 0.25 (S) |
| EASSA-10 | *Salmonella spp.* | Chicken | Germany | > 256 (R) | 4 (S) | 32 (R) | > 64 (R) | 64 (R) | 4 (S) | 0.25 (I) | 0.25 (S) | 1 (S) | 0.03 (S) | > 128 (R) | 0.12 (S) | > 1024 (R) | 128 (R) | 2 (R) | 0.25 (S) |
| EASSA-11 | *Salmonella spp.* | Chicken | Germany | 256 (R) | 4 (S) | 0.25 (S) | 8 (R) | 32 (R) | 8 (S) | 0.25 (I) | 0.25 (S) | 1 (S) | 0.03 (S) | > 128 (R) | 0.12 (S) | > 1024 (R) | 128 (R) | 2 (R) | 0.25 (S) |
| EASSA-12 | *Salmonella spp.* | Chicken | Germany | > 256 (R) | 8 (S) | 2 (S) | 16 (R) | 4 (S) | 8 (S) | 0.5 (I) | 0.25 (S) | 1 (S) | ≤ 0.015 (S) | > 128 (R) | 0.12 (S) | > 1024 (R) | 128 (R) | 4 (R) | 0.25 (S) |
| EASSA-13 | *Salmonella spp.* | Chicken | Germany | 256 (R) | 8 (S) | 0.25 (S) | 16 (R) | 32 (R) | 8 (S) | 0.25 (I) | 0.25 (S) | 1 (S) | 0.03 (S) | > 128 (R) | 0.25 (S) | > 1024 (R) | 128 (R) | 1 (S) | 0.25 (S) |
| EASSA-14 | *Salmonella spp.* | Chicken | Germany | 128 (R) | 4 (S) | 0.25 (S) | 8 (R) | 32 (R) | 4 (S) | 0.5 (I) | 0.5 (S) | 1 (S) | 0.03 (S) | > 128 (R) | 0.25 (S) | > 1024 (R) | 128 (R) | 2 (R) | 0.25 (S) |
| EASSA-16 | *Salmonella spp.* | Chicken | Germany | 128 (R) | 4 (S) | 0.25 (S) | 8 (R) | 16 (R) | 4 (S) | 0.5 (I) | 0.25 (S) | 1 (S) | 0.03 (S) | > 128 (R) | 0.25 (S) | > 1024 (R) | 128 (R) | 1 (S) | 0.25 (S) |
| EASSA-17 | *Salmonella spp.* | Chicken | Germany | 256 (R) | 4 (S) | 0.5 (S) | 16 (R) | 32 (R) | 8 (S) | 0.25 (I) | 0.5 (S) | 2 (S) | 0.03 (S) | > 128 (R) | 0.12 (S) | > 1024 (R) | 128 (R) | 2 (R) | 0.25 (S) |
| EASSA-18 | *Salmonella spp.* | Chicken | Germany | 256 (R) | 4 (S) | 0.25 (S) | 16 (R) | 32 (R) | 8 (S) | 0.25 (I) | 0.5 (S) | 1 (S) | 0.03 (S) | > 128 (R) | 0.12 (S) | > 1024 (R) | 128 (R) | 2 (R) | 0.25 (S) |
| EASSA-19 | *Salmonella spp.* | Chicken | Germany | > 256 (R) | 4 (S) | 1 (S) | 64 (R) | > 64 (R) | 8 (S) | 0.25 (I) | 0.5 (S) | 1 (S) | 0.03 (S) | > 128 (R) | 0.12 (S) | > 1024 (R) | 128 (R) | 2 (R) | 0.25 (S) |
| EASSA-20 | *Salmonella spp.* | Chicken | Germany | > 256 (R) | 16 (S) | > 32 (R) | > 64 (R) | 16 (R) | 32 (R) | 0.5 (I) | 0.5 (S) | 1 (S) | ≤ 0.015 (S) | > 128 (R) | > 512 (R) | > 1024 (R) | 256 (R) | 4 (R) | > 256 (R) |
| EASSA-21 | *Salmonella spp.* | Chicken | Germany | > 256 (R) | 4 (S) | 4 (I) | 32 (R) | 64 (R) | 128 (R) | 0.015 (S) | 0.25 (S) | 1 (S) | ≤ 0.015 (S) | 4 (S) | > 512 (R) | > 1024 (R) | 2 (S) | 0.5 (S) | > 256 (R) |
| EASSA-22 | *Salmonella spp.* | Chicken | Germany | 128 (R) | 4 (S) | 0.25 (S) | 8 (R) | 32 (R) | 4 (S) | 0.5 (I) | 0.25 (S) | 1 (S) | 0.03 (S) | 32 (R) | 0.25 (S) | > 1024 (R) | 128 (R) | 1 (S) | 0.5 (S) |
| EASSA-23 | *Salmonella spp.* | Chicken | Germany | 256 (R) | 4 (S) | 0.5 (S) | 16 (R) | 32 (R) | 8 (S) | 0.5 (I) | 0.5 (S) | 1 (S) | 0.03 (S) | 32 (R) | 0.25 (S) | > 1024 (R) | 128 (R) | 1 (S) | 0.5 (S) |
| EASSA-24 | *Salmonella spp.* | Chicken | Germany | > 256 (R) | 8 (S) | 32 (R) | 64 (R) | 4 (S) | 8 (S) | 0.25 (I) | 0.5 (S) | 1 (S) | ≤ 0.015 (S) | > 128 (R) | 0.25 (S) | 32 (S) | 4 (S) | 1 (S) | 0.06 (S) |
| EASSA-25 | *Salmonella spp.* | Chicken | Germany | > 256 (R) | 8 (S) | 0.25 (S) | 16 (R) | 64 (R) | 8 (S) | 1 (R) | 0.25 (S) | 1 (S) | ≤ 0.015 (S) | 128 (R) | 0.5 (S) | 64 (S) | 4 (S) | 1 (S) | 0.12 (S) |
| EASSA-26 | *Salmonella spp.* | Chicken | Germany | 256 (R) | 8 (S) | 0.25 (S) | 16 (R) | 32 (R) | 16 (I) | 0.25 (I) | 0.5 (S) | 2 (S) | 0.03 (S) | > 128 (R) | > 512 (R) | > 1024 (R) | 128 (R) | 2 (R) | 0.12 (S) |
| EASSA-27 | *Salmonella spp.* | Chicken | Germany | 256 (R) | 4 (S) | 0.25 (S) | 8 (R) | 32 (R) | 8 (S) | 0.25 (I) | 0.25 (S) | 1 (S) | 0.03 (S) | > 128 (R) | 0.12 (S) | > 1024 (R) | 128 (R) | 2 (R) | 0.25 (S) |
| EASSA-39 | *Salmonella spp.* | Chicken | Germany | 256 (R) | 8 (S) | 0.25 (S) | 16 (R) | 32 (R) | 4 (S) | 0.25 (I) | 0.5 (S) | 1 (S) | 0.03 (S) | > 128 (R) | 0.12 (S) | > 1024 (R) | 128 (R) | 2 (R) | 0.12 (S) |
| EASSA-40 | *Salmonella spp.* | Chicken | Germany | 256 (R) | 8 (S) | 0.25 (S) | 8 (R) | 32 (R) | 16 (I) | 4 (R) | 0.25 (S) | 1 (S) | ≤ 0.015 (S) | > 128 (R) | 0.25 (S) | > 1024 (R) | 256 (R) | 4 (R) | 0.25 (S) |
| EASSA-41 | *Salmonella spp.* | Chicken | Germany | 256 (R) | 4 (S) | 0.25 (S) | 8 (R) | 32 (R) | 8 (S) | 0.5 (I) | 0.25 (S) | 1 (S) | ≤ 0.015 (S) | > 128 (R) | 0.12 (S) | > 1024 (R) | 128 (R) | 2 (R) | 0.12 (S) |
| EASSA-56 | *Salmonella spp.* | Chicken | Hungary | > 256 (R) | 8 (S) | 8 (I) | 64 (R) | 2 (S) | 8 (S) | 0.5 (I) | 0.5 (S) | 1 (S) | ≤ 0.015 (S) | > 128 (R) | 0.5 (S) | 64 (S) | 4 (S) | 1 (S) | 0.06 (S) |
| EASSA-59 | *Salmonella spp.* | Chicken | Hungary | > 256 (R) | 8 (S) | 8 (I) | > 64 (R) | 2 (S) | 16 (I) | 0.5 (I) | 0.5 (S) | 1 (S) | ≤ 0.015 (S) | > 128 (R) | 0.25 (S) | 64 (S) | 2 (S) | 2 (R) | 0.06 (S) |
